# Construction of a tumor cell-targeting non-viral gene delivery vector with polyethylenimine modified with RGD sequence-containing peptide

**DOI:** 10.3892/ol.2013.1717

**Published:** 2013-11-29

**Authors:** HAI-BO XING, HONG-MING PAN, YONG FANG, XIAO-YUN ZHOU, QIN PAN, DA LI

**Affiliations:** 1 Department of Intensive Care Unit, Xiasha Hospital, Hangzhou, Zhejiang 310019, P.R. China; 2 Department of Medical Oncology, Sir Run Run Shaw Hospital Affiliated to School of Medicine, Zhejiang University, Hangzhou, Zhejiang 310016, P.R. China

**Keywords:** gene delivery, non-viral vector, polyethylenimine, integrin

## Abstract

The objective of the present study was to construct a novel type of non-viral gene delivery vector with high delivery efficiency and specific tumor cell-targeting ability. The CP9 peptide (CYGGRGDTP) containing Arg-Gly-Asp sequence was employed to be conjugated onto polyethylenimine (PEI) to act as the role of the targeting moiety. The chemical linker, N-succinimidyl-3-(2-pyridyldithio) propionate, was applied during the synthesis of the vector (CP9-PEI). The physicochemical characteristics of the vector were evaluated by the methods of ^1^H-nuclear magnetic resonance, Fourier transform infrared spectroscopy, gel retardation assay, electron microscope observation and particle size detection. HepG2 cells were used to verify the gene delivery efficiency and targeting ability by gene delivery procedure and free CP9 peptide inhibition tests. The results showed that the successful synthesis of CP9-PEI and the synthesized vector may efficiently condense plasmid DNA into round particles with diameters of ~200 nm at a polymer/pDNA ratio of 10. CP9-PEI may deliver the reporter gene into HepG2 cells with higher efficiency and the efficiency may be inhibited by the free CP9 peptide. The present study suggested that the modification of PEI with the CP9 peptide is an effective method to construct a novel tumor cell-targeting non-viral vector, and that the novel vector exhibits great prospect in the field of cancer gene therapy.

## Introduction

Gene therapy has been extensively used for the treatment of various inherited or acquired diseases and has been commonly considered to be the most promising method for cancer therapy over the past decade. The successful manipulation of gene therapy relies on delivery vectors and the selection of functional genes. However, among various considerations for the development of gene therapy techniques that are able to fulfill the requirements of current demands, the lack of safe and efficient gene delivery vectors is a key limiting factor (1). Non-viral vectors, including cationic polymer, have gained increasing interest as alternatives to viral vectors due to their low immune response, low carcinogenic risk and ease of synthesis. However, the greatest disadvantages of non-viral vectors are low efficiency and low specific targeting ability (2). The most attractive strategy to improve transfection efficiency is targeted delivery, where therapeutic genes are delivered to selected cells of interest. A wide range of targeting ligands for cancer gene therapy have been incorporated into non-viral vectors, such as folate, Asn-Gly-Arg and Arg-Gly-Asp (RGD) peptides, transferrins and certain antibodies. Due to the low molecular weight, high specificity and easy availability of peptides, linking peptides may efficiently combine the corresponding receptors on cell surfaces, and it remains an outstanding strategy for improving the delivery efficiency and targeting ability (3). Proteins containing an RGD sequence, together with the integrins that serve as receptors for the sequence, constitute a major recognition system for cell adhesion. The RGD motif has high affinity for αv integrins on tumor cells and has been used for the purpose of actively targeting vectors to deliver drugs or genes into tumor cells (4). Certain studies have adopted RGD-modified polymers or lipids as non-viral vectors for the targeted delivery of genetic material to achieve efficient cancer gene therapy, including antiangiogenic therapy (2,5–7). However, due to the complexity of the characteristics of non-viral vectors, no ideal vectors have been obtained that may have potential for clinical application. The present study attempted to constitute a novel type of vector based on polyethylenimine (PEI; Mw=25 kDa) with conjugation of novel synthesized CP9 peptide (CYGGRGDTP), which exhibits high combination activity with the integrins on tumor cells. The physicochemical characteristics of the novel vector and the biological profiles were studied. The observations of the present study suggested that the targeted delivery of RGD peptides modifies PEI complexes and may be useful for cancer gene therapy.

## Materials and methods

### CP9-PEI synthesis

A total of 5.6 ml (4.5 mg/ml; 1.0×10^−6^ mol resolved in saline solution) PEI solution was reacted with 1.25 mg N-succinimidyl-3-(2-pyridyldithio) propionate (4.0×10^−6^ mol; 1 ml saline and dimethyl sulfoxide solution) at room temperature for 1 h under nitrogen atmosphere protection in the dark with continuous stirring. Once the reaction had finished, 2.8 mg CP9 peptide was slowly dropped into the reaction system for an increased reaction for 2–3 h with the nitrogen protection. The ultimate products were dialyzed against pure running water with dialysis membrane (Mw cut-off, 10,000) for 48 h and then lyophilized for an additional 48 h. The final desiccated white products were stored at −80°C for following experiments.

### ^1^H-nuclear magnetic resonance (NMR) analysis

Synthesized CP9-PEI was dissolved in D_2_O and the ^1^H-NMR spectroscopic data were obtained using a Bruker 400 MHz (Fällanden, Switzerland) spectrometer with eight scans at room temperature.

In total, ~1 mg CP9-PEI was dissolved in pure H_2_O and ultraviolet (UV) detection was performed using a Hitachi U-3400 ultraviolet spectrophotometer (Hitachi, Ltd., Tokyo, Japan).

### Fourier transform infrared spectroscopy (FTIR) detection

A total of ~1 mg CP9-PEI was prepared by dispersing with potassium bromide and the complexes were compressed into disks. The FTIR detection was conducted on a FTIR spectrometer (Spectrum 2000; Perkin-Elmer, Waltham, MA, USA). Overall, 16 scans were signal averaged to a resolution of 2 cm^−1^ at room temperature.

### Gel retardation assay

Electrophoretic mobility of the polymer CP9-PEI/plasmid DNA (pDNA) polyplexes was measured using an agarose gel electrophoresis system (Invitrogen, Carlsbad, CA, USA). An appropriate amount of CP9-PEI was added to an equal volume of pDNA solution to achieve the desired polymer/pDNA ratio (N/P ratio). In total, 180 μl CP9-PEI/pDNA solutions (N/P ratios equal to 0, 0.5, 1, 2, 3, 4 and 5) were loaded into the loading wells of the agarose gel. The gel electrophoresis was performed at room temperature in Tris-acetate-EDTA buffer in 1% (w/w) agarose gel at 80 V for 45 min. The DNA bands were visualized by an UV illuminator (IS-1000; Alpha Innotech, San Leandro, CA, USA).

### Transmission electron microscope observation

The CP9-PEI/pDNA solution was prepared (N/P ratio of 10) using saline as the solvent for transmission electron microscope observation. The mixture solution was vortexed for 1 min and left standing for 20 min, then ~1 ml solution was dropped on the copper net. The samples were dried and the observation of the morphology of the polyplexes was conducted under a JEM-2010 transmission electron microscope (JEOL, Tokyo, Japan).

As with the analysis of particle size, a series of CP9-PEI/pDNA solutions (N/P ratios of 5, 10, 20 and 30) were prepared in saline and analysis was performed using a 90 Plus particle size analyzer (Brookhaven Instruments Corporation, Holtsville, NY, USA) at 25°C. Scattering light was detected at 90° and the wavelength was 670 nm.

### In vitro gene delivery

The HepG2 cells (ATCC, Rockville, MD, USA) were seeded in 48-well plates at a density of 2.5×10^4^/well with 850 μl Dulbecco’s modified Eagle’s medium (DMEM) containing 10% fetal calf serum (FCS) at 37°C for 24 h culture. When the confluence of the cells had reached 70–80%, the culture medium was replaced with 800 μl serum-free DMEM and the polyplexes of CP9-PEI/pDNA with various N/P ratios (5, 10, 20 and 30) containing 1 μg pCMV-luc were dropped into each well (PEI was used as the polymer control). The polyplexes were incubated with the cells for 6 h at 37°C, followed by supplementation with DMEM containing 10% FCS for an additional 36 h. Later, the incubation medium were removed and the cells were rinsed with phosphate-buffered saline (PBS) and frozen-thawed in 200 μl PBS at −80°C. The luciferase activity of the cell extracts was measured by a luciferase assay system (Promega Corporation, Madison, WI, USA). The quantity of total protein per well of cell extracts was determined by protein assay kit (BCA; Pierce Biotechnology, Inc., Rockford, IL, USA).

### Inhibition effect

The gene delivery process was similar to that of *in vitro* gene delivery. However, in this step, prior to the addition of polyplexes, the free CP9 peptide at different concentrations (10, 50 and 100 nmol/l) was first added into the culture system for 2 h of co-culture with the cells [peptide containing RGE sequence (CYGGRGETP) acted as control group] and then the gene delivery process was continued.

### Statistical analysis

Unless noted otherwise, results from *in vitro* experiments are represented by at least three independent experiments. All data are expressed as the mean ± standard deviation. Statistical analysis was performed using one-way analysis of variance and Fisher’s least significant difference test. Analysis was performed using SPSS 12.0 (SPSS, Inc., Chicago, IL, USA). P<0.05 was considered to indicate a statistically significant difference.

## Results

### ^1^H-NMR analysis

**Figure 1:**
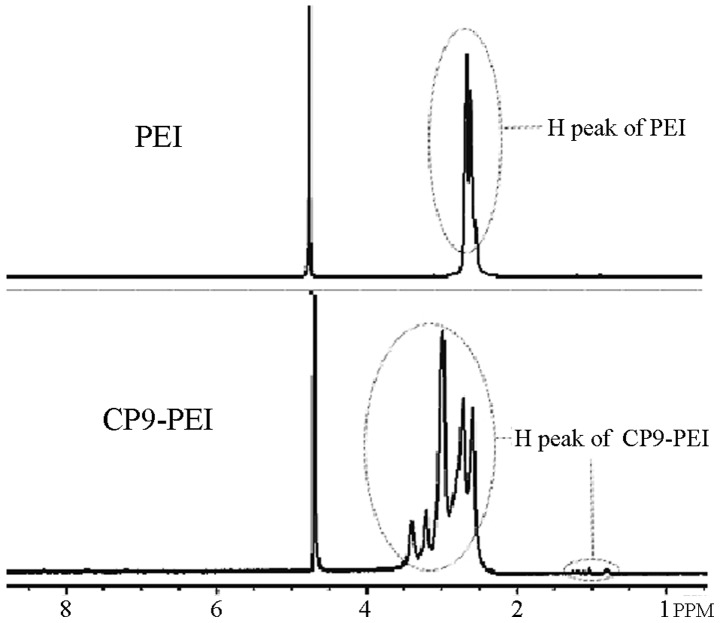
^1^H-NMR analysis of PEI and CP9-PEI. ^1^H-NMR, ^1^H-nuclear magnetic resonance; PEI, polyethylenimine.

The results of the ^1^H-NMR analysis (Fig. 1) showed that as with PEI, the main three chemical displacements of H proton were located between 2.1 and 3.0 ppm, which correspond with the three H protons in the structure of PEI. However, when the CP9 peptide was conjugated onto PEI, new H proton waves were identified between the displacements of 3.0 and 3.5 ppm, and the superposition waves appeared between the range of 2.1 and 3.0 ppm.

### FTIR detection

**Figure 2:**
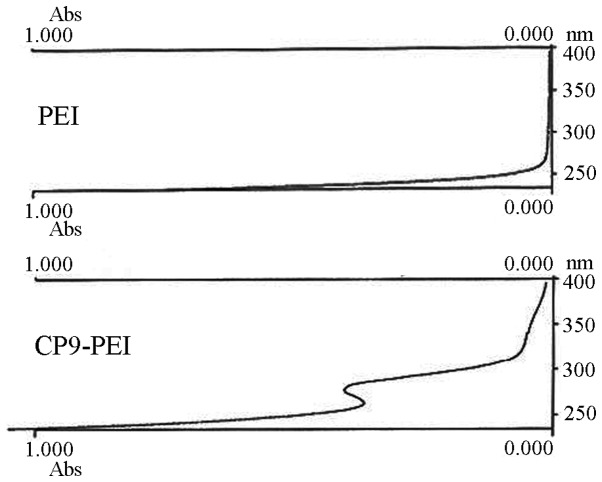
UV detection of PEI and CP9-PEI. UV, ultraviolet; PEI, polyethylenimine.

The FTIR detection (Fig. 2) showed that at wavelengths of 3,420, 2,925 and 2,852 cm^−1^, PEI and CP9-PEI exhibited absorbance peaks that showed the radical of -NH or -CH_2_ in their chemical structures. However, a new absorbance peak at 1,630 cm^−1^ appeared, which suggested carbonyl group (C=O) formation in the synthesized CP9-PEI.

### Gel retardation action

**Figure 3:**
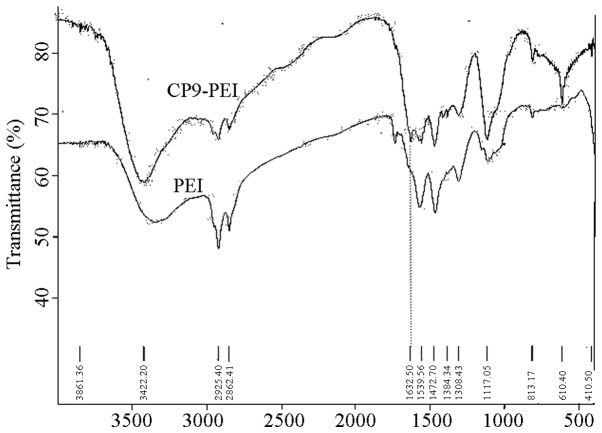
FTIR analysis of PEI and RGD peptide-PEI. FTIR, Fourier transform infrared spectroscopy; PEI, polyethylenimine; RGD, arginine-glycine-aspartic acid.

The gel retardation assay showed that CP9-PEI may completely retard the mobility of the plasmids in the agarose gel at the N/P ratio of 4; while in PEI, the N/P ratio was 3 (Fig. 3).

### Polyplex particle size and morphology

**Figure 4:**
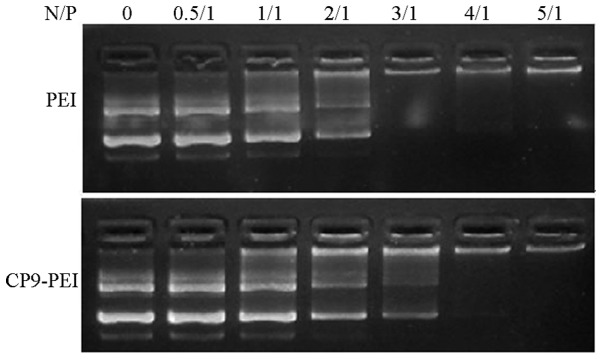
Gel retardation assay of PEI and CP9-PEI. PEI, polyethylenimine.

**Figure 5:**
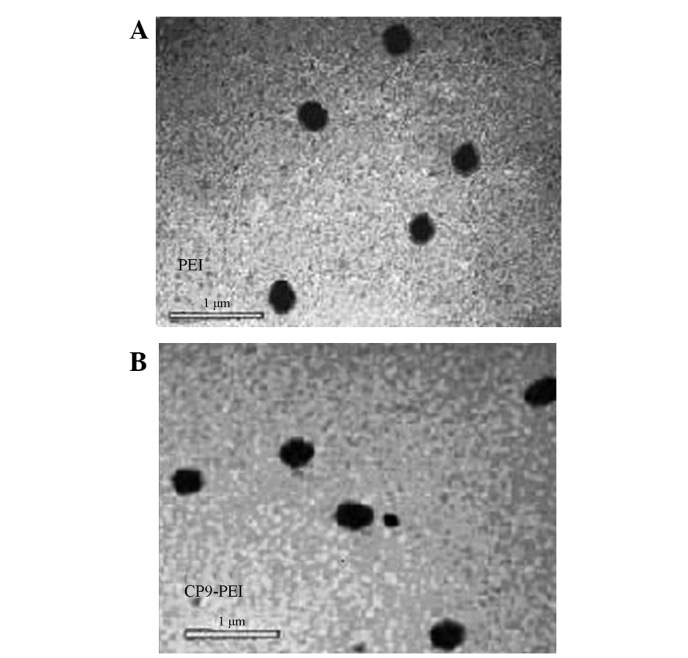
Formation of PEI-pCMV-luc or CP9-PEI-pCMV-luc polyplexes detected by transmission electron microscopy (magnification, ×10,000). PEI, polyethylenimine.

By transmission electron microscopy (Fig. 4), the nanoparticles comprised with CP9-PEI or PEI plasmids showed round or round-like compact particles with diameters of ~200 nm when the N/P ratio was 10. Further detection (Fig. 5) exhibited that in CP9-PEI or PEI plasmids, the diameter of the particles decreased with an increased N/P ratio from 5 to 30. However, at an N/P ratio of 10, the diameter of the CP9-PEI/pCMV-luc polyplexes was ~187.54±13.14 nm, which was similar to that of the PEI/pCMV-luc polyplexes (201.01±11.22 nm; P=0.248).

### Gene delivery

**Figure 6:**
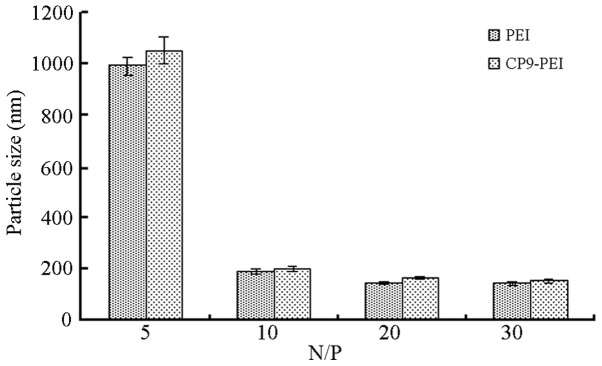
Particle size assay of PEI-pCMV-luc and CP9-PEI-pCMV-luc. PEI, polyethylenimine; N/P, polymer/pDNA ratio.

The outcome of the gene delivery of the vectors in HepG2 cells (Fig. 6) suggested that CP9-PEI and PEI reached the highest delivery efficiency at an N/P ratio of 10. However, under that condition, the efficiency of CP9-PEI was 3.98×10^8^ RLU/mg protein, but that of PEI was only 1.95×10^8^ RLU/mg protein.

### Inhibition effect

At an N/P ratio of 10, the inhibition experiments showed that with the increasing concentration of free CP9 peptides (from 0 to 100 nmol/l), the delivery efficiency of CP9-PEI decreased from 3.98×10^8^ to 1.80×10^8^ RLU/mg protein. However, as with the control peptides containing RGE sequence, no such inhibition effect was identified.

## Discussion

Numerous developments in the field of gene therapy have been made and >1,800 programs have entered clinical tests (8). Among these, viral vectors were predominantly employed. However, the viral vectors exhibit a number of shortcomings, such as low hereditary material carrying capability, high immunogenicity and safety considerations, including carcinogenesis. Previous studies of non-viral vectors, including liposomes and cationic polymers, have recently appeared and have obtained great achievements (1,8).

PEI, a chemical with multiple amine structures, is currently a ‘golden standard’ in the field of non-viral gene delivery for its outstanding characteristics, including high delivery efficiency, simple structure, ease of preparation, good value and safety. In 1995, Boussif *et al* developed the gene delivery function of PEI (9) and it has since become the focus for research. Certain strategies have been developed to modify PEI to improve its efficiency and avoid the disadvantages, such as non-specific cell targeting ability (10). Some factors may affect the delivery profiles of PEI, including the molecular weight, branch structures, menstruum system and the parameters of the delivery process (11). The majority of previous studies has shown that the 25-kDA branched PEI is a rather prospective non-viral delivery reagent in gene therapy (12–14). The present study adopted the 25-kDa branched PEI as the backbone for constructing the novel vector to endow more beneficial characteristics.

The current study employed the CP9 ligand peptides, which contain the RGD sequence and may combine efficiently with the integrins on the majority of tumor cells, to construct the novel vector. Since the ligand-receptor integration mechanism can switch on the receptor-mediated delivery pathway, such construction strategy must endow a high-efficiency vector and theoretically, a tumor cell-targeting function (15).

In the current study, ^1^H-NMR, UV detection and FTIR methods were first utilized to confirm the successful synthesis of CP9-PEI. Only the CP9 moiety was victoriously conjugated onto the primary amines of PEI and new H proton peaks appeared on the spectrum of ^1^H-NMR, peptide absorbance emerged at ~270 nm on the UV detection and new carbonyl coupling (C=O) produced a peak of 1,630 cm^−1^ on the spectrum of FTIR (Figs. 1–3). Evidently, the 1,630 cm^−1^ peak was affected by the existence of abundant amine groups in PEI (16). However, the that peak formed at 1,630 cm^−1^ was small, which must be imputed to the low conjugation ratio of CP9 to PEI.

One the most critical steps of non-viral vector delivery function is its plasmid condensing ability (17). Only when nanoparticles of the polymer/plasmids form may the vectors enter the cells and nucleus, releasing the hereditary material (18). The results of the present study showed that CP9-PEI may completely condense the plasmids at a N/P ratio of 4 (Fig. 4). However, in PEI, the responding N/P ratio was 3, which suggested that with conjugation, the moiety of CP9 and the condensing ability were impaired. However, such impairment had little influence, since at a N/P ratio of 10, CP9-PEI condensed the plasmids into nanoparticles with diameters of ~200 nm (Fig. 5). The condensing process was affected by certain factors, including the types of polymers, molecular weight, modification of polymers and menstruum system (19). Considering the limitation of endocytosis of the vector, the Brown movement and the precipitation of the nanoparticles onto the cells surface, it is generally considered that the optimal diameter of polyplexes for gene delivery is 100–300 nm (20). In the present study, the synthesized CP9-PEI showed optimal characteristics, which have been determined as prospective profiles for excellent vectors (Fig. 6). Other factors, including molecular weight, modification of polymers and menstruum system, remain under investigation.

**Figure 7:**
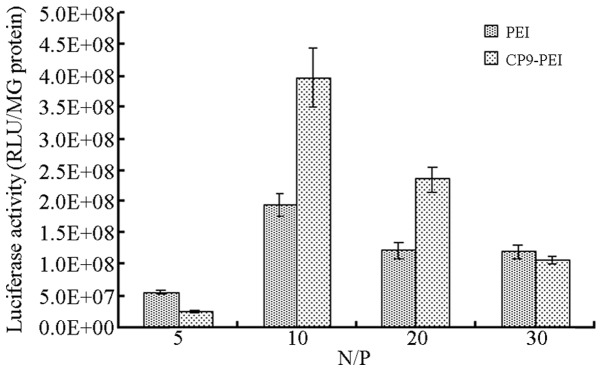
Gene delivery results of PEI and CP9-PEI in HepG2 cells. PEI, polyethylenimine; N/P, polymer/pDNA ratio.

**Figure 8:**
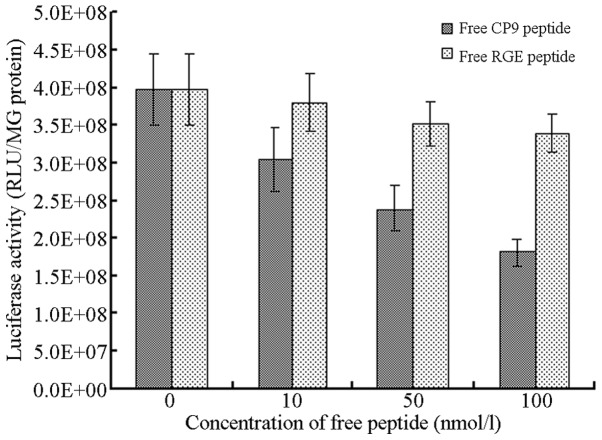
Inhibition effect of free RGD peptide on the transfection efficiency of CP9-PEI in HepG2 cells. PEI, polyethylenimine; RGD, arginine-glycine-aspartic acid.

The ligand conjugation strategy may initiate the receptor-mediated gene delivery process, which replaces part of the static electricity-mediated pathway between polymers and cells (21). Such a replacement effect may endow the vector-specific receptors targeting capability and reduce non-specific contact delivery function. Certain types of conjugation ligands have been previously studied, such as epidermal growth factor, transferrin and monoclonal antibodies (2). In general, the conjugation of ligands effectively improves the level of delivery efficiency of the vector through switching on the receptor-mediated delivery pathway and also renders the vector more advantageous with targeting properties. The αvβ3/αvβ5 types of integrin are excellent targets for cancer gene therapy, as they exhibit markedly higher expression levels in numerous types of cancer cells, such as HepG2, and in endothelial cells in tumor angiogenesis (22). The main function of integrin is its involvement in the procedure of cancer cell adherence, invasion and metastasis. Commonly, the short peptides containing the RGD sequence (RGDS) have been considered to exhibit great combination activity with integrin, for example, the RGDS peptides have been confirmed to simulate the characteristics of the natural ligand of integrin (23). The results of the current study (Fig. 7) showed that while conjugated with the CP9 peptides, PEI exhibited more than two-fold the delivery efficiency compared with that of pure PEI. Furthermore, the free peptide inhibition tests, including the RGE sequence-containing peptide tests, suggested that the improved effect was due to the RGD core sequence (Fig. 8). Evidently, the CP9 moiety improved the efficiency and simultaneously led to the integrin targeting capability. Integrin was widely expressed in the majority of types of cancer cells at a higher level and it may be speculated that the CP9-PEI vector has vast prospects in cancer gene therapy.

The main aim of the present study of non-viral vectors was to investigate their potential for application *in vivo*. Currently, the majority of studies analyzing PEI have focused on the local application, since the positive charge of the polyplex remains a great obstacle for its systemic employment. Certain strategies, including PEGylation, have been previously studied (24). In conclusion, the new CP9-PEI vector synthesized in the current study exhibited optimal characteristics, enhanced gene delivery efficiency and tumor cell-targeting capability. It has been considered that with increased modification, such as PEGylation, the vector may be developed into an ideal carrier for gene therapy.
